# Impact of video -based incentive on patient willingness to read the informed consent form with comprehension before minimally-invasive surgery: a randomized controlled trial

**DOI:** 10.20452/wiitm.2025.17957

**Published:** 2025-06-05

**Authors:** Weronika Kisielewska, Michał Kościołek, Kryspin Mitura, Laura Kacprzak, Małgorzata Pajer, Krystian Kisielewski, Bernard Mitura, Weronika Kowalczyk

**Affiliations:** Faculty of Medical and Health Sciences, University of Siedlce, Siedlce, Poland; Department of General Surgery, Siedlce Hospital, Siedlce, Poland; Third Department of Psychiatry, Institute of Psychiatry and Neurology, Warszawa, Poland; Faculty of Medicine, Jagiellonian University Medical College, Kraków, Poland; Faculty of Medicine, Medical University of Warsaw, Warszawa, Poland

**Keywords:** anxiety, informed consent, laparoscopy, surgery, video

## Abstract

**INTRODUCTION:**

The purpose of the informed consent (IC) form is to enable patients to make a conscious choice based on complete and comprehensible information about a planned surgical procedure, its risks, benefits, and alternative treatment methods. Obtaining information about other therapeutic options is particularly important in the case of laparoscopic surgeries which are becoming increasingly popular. Unfortunately, as the literature indicates, most patients do not read the IC form.

**AIM:**

The aim of this study was to evaluate the impact of video material on encouraging patients to thoroughly read the IC form.

**MATERIALS AND METHODS:**

This parallel design study comprised 102 patients referred for elective laparoscopic surgical procedures. The participants were randomized in a 1:1 ratio. The block randomization consisted of alternating, weekly assignment of patients to the intervention (video) group and the control group. Anxiety levels were evaluated using a translated version of the Amsterdam Preoperative Anxiety and Information Scale (APAIS).

**RESULTS:**

The patients in the video group were more likely to read the entire IC form (83.67%) than those in the control group (33.96%; P = 0.003). However, according to the APAIS scale, reading the IC form had no impact on the level of anxiety (P = 0.72) and information demand (P = 0.9). The most frequently given reason for not reading the IC form was its excessive length (32.61% of the responses).

**CONCLUSIONS:**

Video materials demonstrate a remarkable potential in enhancing the awareness of the IC process importance and should be increasingly implemented into everyday medical practice.

## INTRODUCTION

Informed consent (IC) prior to surgery constitutes a fundamental element of the treatment process, serving not only as a legal requirement but also as an ethical corner-stone in the relationship between a patient and a medical practitioner.^1^^,^^2^ A patient’s right to receive information about alternative treatment methods, including different surgical approaches, is crucial.^3^ Surgeons are therefore obliged to inform patients about available therapeutic options, outlining their benefits, disadvantages, potential risks, and the implications of choosing a specific approach or declining treatment. This obligation is particularly pertinent in surgical procedures, where an array of different techniques may result in significant variations in complication rates. In contemporary practice, such differences are especially evident between conventional open surgery and laparoscopic methods. Minimally-invasive surgery (MIS) has gained widespread popularity popularity due to numerous advantages, including reduced postoperative pain, shorter recovery time, and a lower risk of complications in comparison with conventional open surgery.^4^^,^^5^. However, laparoscopic procedures may also carry unique complications.^6^ As IC forms typically include descriptions of various surgical approaches and associated risks, it is vital for patients awaiting MIS to thoroughly read them. Unfortunately, as indicated in previous studies, ^7^^,^^8^ most patients do not read the IC form or read it partially. This lack of engagement raises concerns about whether patients understand the provided information, highlighting the need for clearer and more appealing ways of conveying important details. Moreover, IC constitutes one of the most significant elements in alleviating patient anxiety and, according to evidence,^9^ patients who do not receive enough information experience a higher level of anxiety, are less satisfied, and have trouble making a decision. The advancements in modern technologies, such as the ease of creating video materials and the use of interactive platforms, bring new opportunities for patient education and improvements in the IC process.^10^^,^^11^

**Figure 1:**
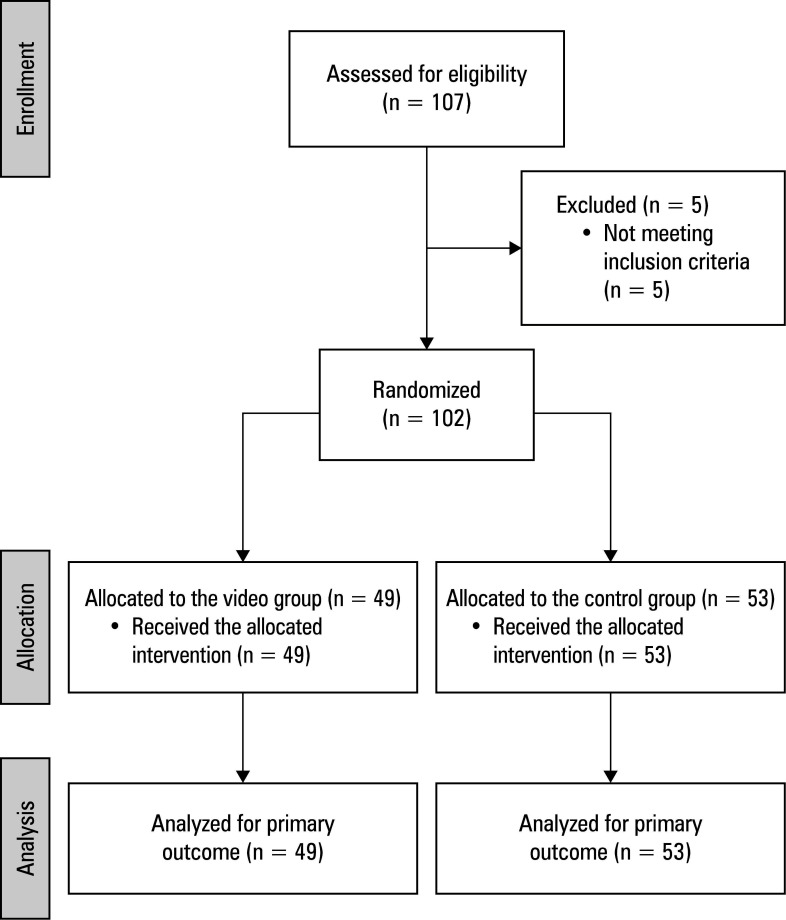
CONSORT 2025 flow diagram^13^

## AIM

Although a range of solutions aimed at in-creasing the legibility of the IC document is avail-able in the literature^12^ to our knowledge, this is the first study designed to assess the impact of a video-based intervention as an incentive to read the IC form. The secondary objective of our study was to evaluate differences in anxiety levels and information demand between the individuals who meticulously read the document and those who did not. Furthermore, we aimed to identify the underlying reasons for patients’ lack of interest in the IC form, and gather their subjective opinion regarding the adequacy and comprehensiveness of the information provided therein.

### Materials and Methods

The study comprised 102 patients scheduled for elective laparoscopic surgery at the General Surgery Department of the Siedlce Hospital between May and December 2024. Adult patients, rated with the American Society of Anesthesiologists (ASA) physical status classification as class I to III, who could un-derstand the video and the information contained in the questionnaires in their native language were considered eligible. Patients with mental dis-orders or unable to complete the questionnaire were excluded from the study. The participants whose demographic variables, surgery-related factors, or questionnaire responses could not be assigned to specific categories were also includ-ed in the statistical analysis. If the questionnaire was incomplete, the patient was excluded from the study. The study had a parallel group design. The participants were randomized in a 1:1 ratio into the video group (VG) and the control group (CG). The block randomization method consisted of alternating, weekly assignment of patients to the groups. The block size was determined by the number of patients undergoing laparoscopic procedures during a given week and, at the end of the study, the missing group was completed by repeating the blocks. The patients were not informed about their assignment to a specific group or the factors determining their allocation. Only the researchers responsible for data collection were not blinded to the randomization process. All patients who voluntarily agreed to participate in the study completed a questionnaire assessing anxiety and information demand upon admission to the hospital. Patients in the VG watched a short video designed to encourage them to fully familiarize themselves with the IC document. A few hours later, they were asked to fill in a questionnaire evaluating whether they read the entire IC form. If the answer was negative, they were asked to indicate reasons for it. Then, the patients filled out the anxiety assessment questionnaire once again. Finally, the participants were asked to complete a questionnaire regarding the information contained in the IC document. This included answering questions using a 5-point Likert scale, aimed at evaluating their subjective opinions on how well they believed to have been in-formed about the surgical procedure. There was no patient or public involvement in the study de-sign, course, and reporting. The study was concluded upon the decision of the principal investigator after reaching the target sample size of 100 participants. The manuscript was prepared in accordance with the CONSORT 2025 guidelines.^13^

**TABLE 1:** Characteristics of the video group and control group

Parameter		Video group (n = 49)	Control group (n = 35)	P value
Age, y, mean (SD)		52.08 (13.08)	53.87 (14.94)	0.1
Age group, y	18–44	12 (24.49)	19 (35.85)	0.09
	45–64	26 (53.06)	17 (32.08)	
	³65	11 (22.45)	17 (32.08)	
Sex	Women	26 (53.06)	37 (69.81)	0.08
	Men	23 (46.94)	16 (30.19)	
ASA class	I	10 (20.41)	11 (20.75)	0.79
	II	27 (55.1)	27 (50.94)	
	III	12 (24.49)	14 (26.42)	
	Not specified	0	1 (1.89)	
Previous surgery	Yes	40 (81.63)	38 (71.7)	0.3
	No	9 (18.37)	15 (28.3)	
Type of surgery	Hernia surgery	18 (36.73)	15 (28.3)	0.36
	Cholecystectomy	31 (63.27)	38 (71.7)	
Education	Primary	3 (6.12)	7 (13.21)	0.3
	Secondary	18 (36.73)	25 (47.17)	
	Higher	22 (44.9)	17 (32.08)	
	Not specified	6 (12.24)	4 (7.55)	
Marital status	Single	6 (12.24)	7 (13.21)	0.99
	Married	36 (73.47)	38 (71.7)	
	Divorced	1 (2.04)	1 (1.89)	
	Widowed	6 (12.24)	7 (13.21)	
Population of the city of residence, n	<10 000	26 (53.06)	23 (43.4)	0.5
	10,000–49,000	4 (8.16)	3 (5.66)	
	50 000–100 000	16 (32.65)	25 (47.17)	
	>100 000	3 (6.12)	2 (3.77)	

Data are presented as numbers and percentages unless indicated otherwise.

Abbreviations: ASA, American Society of Anesthesiologists

### Video material

Our team created a video lasting 1 minute and 52 seconds, aimed at raising patients awareness of the importance of the IC process and encouraging them to fully familiarize with the IC document. The video presents key issues included in the IC form, such as a description of the underlying disease, proposed treatment, alternative therapeutic methods, consequences of foregoing treatment, and risks associated with surgery. Patients in the VG watched the video in a quiet, isolated screening room upon admission to the Department. The video is available at: https://www.youtube.com/watch?v=mm-50OpQ2n8.

### Anxiety measuring tool 

The Amsterdam Preoperative Anxiety and Information Scale (APAIS) enables the assessment of patient anxiety level and their need for information related to a surgical procedure. It contains 6 questions rated on a Likert scale, ranging from 1 (not at all) to 5 (extremely), allowing patients to express the intensity of their anxiety or information requirements. The preoperative anxiety subscale scores ranged from 4 to 20 points, whereas the need-for-information subscale scores spanned from 2 to 10 points. Results in the range of 2–4 points indicated a low demand for information about the operation, 5–7 points corresponded to a medium demand, and 8–10 points translated into high demand. A score above 11 on the anxiety subscale meant that the patient was experiencing anxiety.

### Statistical analysis

The sample size was determined based on the results of previous studies, taking into account the mean (SD) for the collected data.^14^ The formula used to calculate the minimum sample size was as follows: minimum sample size = 2 × (Z_(1/2α)_ + Z_β_)² × SD² / (μ_T_ – μ_R_)^2, ^where Z_(1/2α)_ is a value from the normal distribution corresponding to the significance level α (for α = 0.05, Z = 1.96); Z_β_ is a value from the normal distribution corresponding to the test power (1 – β); SD is the standard deviation of the measured variable; and μ_T_ − μ_R_ is the difference in means between the test group (T) and the reference group (R). In this case, the required number of participants in each group was 41, with *P* = 0.05 (Z_(1/2α)_ = 1.96), the test power of 0.95 (Z_β_ = 1.645), and an assumed difference of (μ_T_ – μ_R_)^2^ = 3.75, based on total APAIS results in both groups. SD used was 6.46. To address potential dropouts, 102 participants who met the inclusion and exclusion criteria were enrolled in the study and included in the analysis. The study included an exploratory comparison.

All calculations were performed using STATISTICA 13.3 software 2023 (Tibco Software Inc., Santa Clara, California, United States). The Shapiro–Wilk test was used to assess the distribution of quantitative variables. The *t* test and the Mann–Whitney test were used for comparison of continuous variables, presented as mean (SD) or median (interquartile rage [IQR]), respectively. The χ² test (with the Yates correction for small groups [n <⁠10]) and the Fisher exact test (n <⁠5) were used to compare categorical data, presented as counts and percentages. For dependent samples, the Wilcoxon signed-rank test was used to compare the values obtained on admission and those registered a few hours later. A *P* value below 0.05 was considered significant.

### Ethics

The study was approved by the Regional Bioethical Committee at the University in Siedlce (7/2024). Written IC was obtained from all the participants. This study adhered to the principles of the 1964 Declaration of Helsinki and its subsequent amendments.

## RESULTS 

The study originally enrolled 107 patients, but 5 were excluded because they did not meet the inclusion criteria. Finally, the study comprised 102 patients, 49 of whom were as- signed to the VG and watched the video and 53, to the CG Figure 1 . All patients successfully completed the study and adhered to the instructions. The VG filled out the questionnaires and watched the video material. No adverse events were identified.

**TABLE 2:** The relationship between demographic and surgery‑related factors and patients’ reading of the consent form

Parameter	Video group (n = 49)		P value	Control group (n = 53)		P value
	Read completely (n = 41)	Did not read or read partially (n = 8)		Read completely (n = 18)	Did not read or read partially (n = 35)	
Age, y, median (IQR)	52 (43.5–61.5)	48.5 (45.25–68.75)	0.99	50.5 (40–56.25)	56 (41–70)	0.19
Age group, y						
18–44	11 (26.83)	1 (12.5)	0.69	7 (38.89)	12 (34.29)	0.18
45–64	21 (51.22)	5 (62.5)		8 (44.44)	9 (25.71)	
≥65	9 (21.95)	2 (25)		3 (16.67)	14 (40)	
Sex						
Women	21 (51.22)	5 (62.5)	0.71	14 (77.78)	23 (65.71)	0.53
Men	20 (48.78)	3 (37.5)		4 (22.22)	12 (34.29)	
ASA class						
I	10 (24.39)	0	0.27	8 (44.44)	3 (8.57)	0.01
II	21 (51.22)	6 (75)		6 (33.33)	21 (60)	
III	10 (24.39)	2 (25)		4 (22.22)	10 (28.57)	
Not specified	0	0		0	1 (2.86)	
Surgery history						
Yes	33 (80.49)	7 (87.5)	0.85	7 (38.89)	31 (88.57)	<0.001
No	8 (19.31)	1 (12.5)		11 (61.11)	4 (11.43)	
Type of surgery						
Hernia surgery	15 (36.59)	3 (37.5)	0.99	3 (16.67)	12 (34.29)	0.18
Cholecystectomy	26 (63.41)	5 (62.5)		15 (83.33)	23 (65.71)	
Education						
Primary	3 (7.32)	0	0.52	2 (11.11)	5 (14.29)	0.86
Secondary	16 (39.02)	2 (25)		25 (50)	16 (45.71)	
Higher	18 (43.9)	4 (50)		5 (27.78)	12 (34.29)	
Not specified	4 (9.76)	2 (25)		2 (11.11)	2 (5.71)	
Marital status						
Single	5 (12.2)	1 (12.5)	0.65	3 (16.67)	4 (11.43)	0.55
Married	29 (70.73)	7 (87.5)		14 (77.78)	24 (68.57)	
Divorced	1 (2.44)	0		0	1 (2.86)	
Widowed	6 (14.63)	0		1 (5.56)	6 (17.14)	
Population of the city of residence, n						
<10 000	23 (56.1)	3 (37.5)	0.71	8 (44.44)	15 (42.86)	0.39
10 000–49 000	3 (7.32)	1 (12.5)		0	3 (8.57)	
50 000–99 000	13 (31.71)	3 (37.5)		10 (55.56)	15 (42.86)	
>100 000	2 (4.88)	1 (12.5)		0	2 (5.71)	
APAIS score, median (IQR)						
APAIS anxiety, points	12 (7–15.5)	14 (9.25–15.75)	0.38	16 (14.75–19.25)	12 (8–14)	<0.001
APAIS information, points	5 (4–8)	4 (2.5–5)	0.09	8 (7–10)	5 (4–7)	<0.001
APAIS total, points	18 (12–21)	18.5 (13.5–20.75)	0.73	23.5 (22–29.25)	18 (13–20)	<0.001

Data are presented as numbers and percentages unless indicated otherwise.

Abbreviations: APAIS, Amsterdam Preoperative Anxiety and Information Scale; IQR, interquartile range; others, see TABLE 1

There were no differences between the groups in terms of demographic information and surgical characteristics TABLE 1.

TABLE 2 details the differences between the individuals who read the IC form and those who either did not read it or read it partially, separately for each group. In the CG, differences in terms of surgical history (P <0.001) and ASA status (P = 0.01) were observed. Most patients with no history of previous surgery read the IC form (11/15 [61.11%]), whereas those who had undergone surgery in the past either did not read it or read it only partially (31/38 [88.57%]). In the CG, no differences were found in terms of age (P = 0.19), sex (P = 0.53), marital status (P = 0.55), type of surgery (P = 0.18), level of education (P = 0.86), or the population of the city of residence (P = 0.39) between the patients who read the IC form completely and those who did not. In the VG, we did not observe any differences between the individuals who read the IC document and those who did not, in terms of all the abovementioned factors. In the CG, the patients who completely familiarized themselves with the IC statement exhibited higher values of anxiety (median [IQR], 16 [14.75–19.25] vs 12 [8–14]; P <0.001) and information demand (median [IQR], 8 [7–8] vs 5 [4–70; P <0.001) on the APAIS scale, as compared with patients who did not read the IC form. 

**FIGURE 2:**
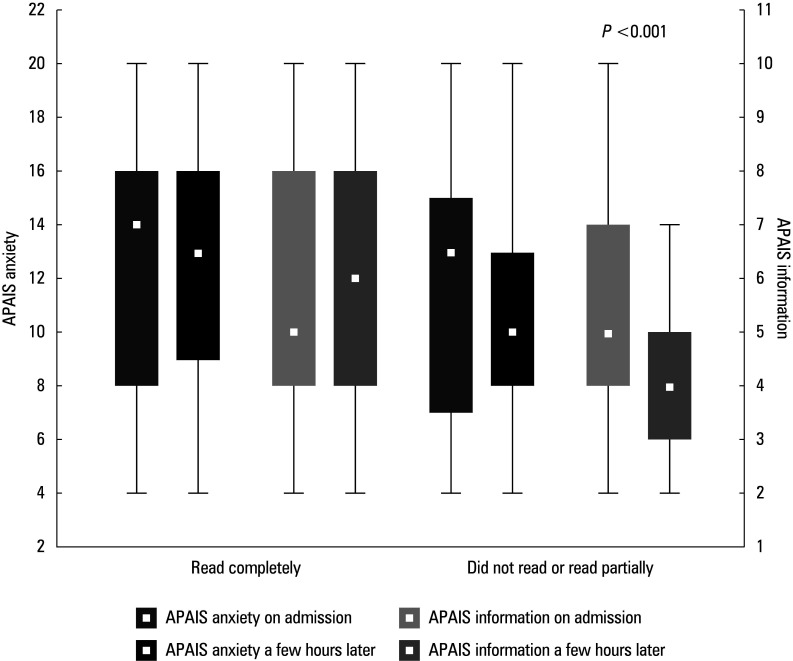
Amsterdam Preoperative Anxiety and Information Scale anxiety and information scores on admission and a few hours later; a comparison between the patients who read the consent form and those who did not. The results are presented as median (IQR). Whiskers indicate the minimal and maximal values, boxes indicate the IQR, and white squares represent the median value.

Abbreviations: see TABLE 2

**FIGURE 3:**
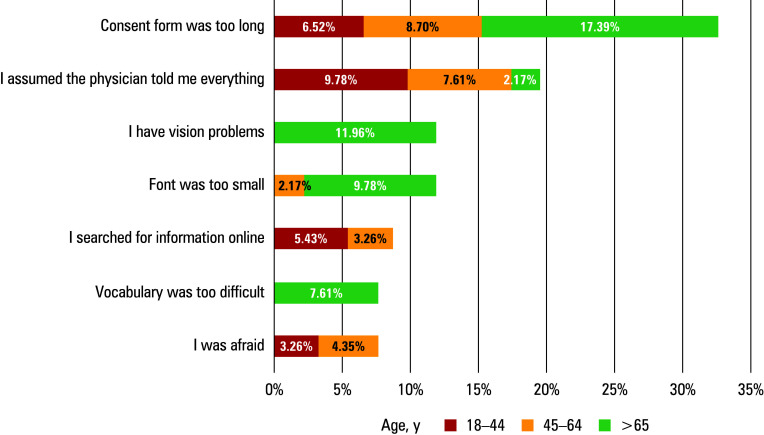
Reasons why the patients did not read the consent form. Data in the boxes indicate the percentage of responses to each question, categorized by age groups.

In the VG, there were no differences between the individuals who read the IC form and those who did not, in terms of anxiety level (P = 0.38) and the need for information (P = 0.09). The patients in the VG were more likely to read the IC form completely (83.67%) than those in the CG (33.96%; P = 0.003), while partial reading or failure to read was more frequent in the CG (66.04% vs 16.33%; P <0.001). The level of anxiety and the need for information essentially did not change among the individuals who read the IC form between the moment of admission (median [IQR], 14 [8–16] vs 13 [9–16]; P = 0.72) and a few hours later (median [IQR], 5 [4–8] vs 6 [4–8]; P = 0.9). In the group of participants who did not read document, however, a decrease in both values was observed (median [IQR], 13 [7–15] vs 10 [8–13]; P = 0.07 and median [IQR], 5 [4–7] vs 4 [3–5]; P <0.001 for anxiety and information demand, respectively; FIGURE 2). The total number of responses indicating the reasons for not reading the IC form was 92, with each patient having the option to select multiple reasons. The most frequently given reason for not reading the IC document was its excessive length, accounting for 30 out of 92 responses (32.61%), especially notable in the oldest age group. The second most common reason was the assumption that all the information had been provided by the physician (18/92 [19.56%]). The fewest responses concerned anxiety and difficulties understanding the vocabulary, constituting 7/92 responses (7.61%; FIGURE 3).

**FIGURE 4:**
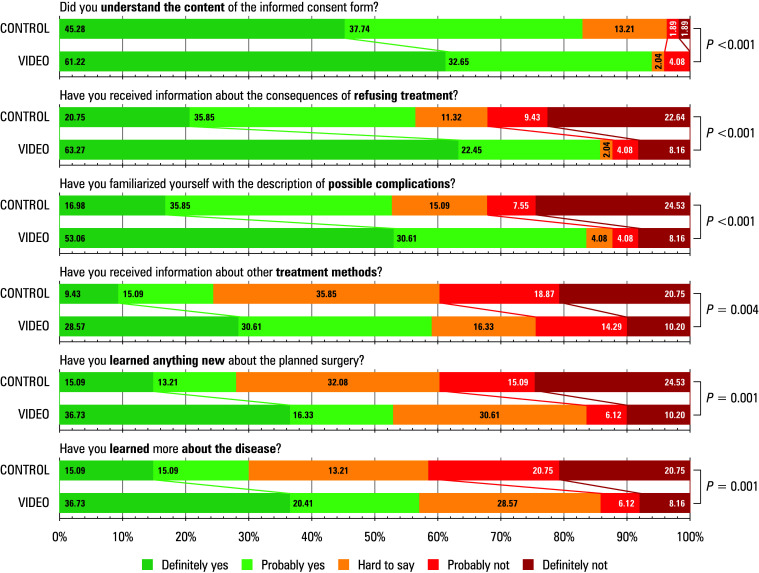
Patients’ subjective opinion on the extent of the information obtained. Data in the boxes indicate the percentage of responses to each question according to a 5‑point Likert‑scale.

While evaluating patient responses regarding the level of information obtained, we observed better outcomes in the VG than the CG. Detailed results are presented in FIGURE 4.

## DISCUSSION

In modern MIS, there are 2 critical aspects concerning the content of IC forms. Firstly, some medical centers still do not offer the basic range of laparoscopic procedures, resulting in patients being subjected to conventional open surgeries, even in the case of common procedures.^15^ Still, some of these patients might be unaware of the availability of minimally-invasive approaches at other facilities. Secondly, specialized centers employing MIS techniques may encounter complications that are absent in conventional open surgery.^16^ These issues should always be addressed in IC forms given to patients for a comprehensive reading before each surgical intervention in all health care centers. The findings of our research are consistent with previous studies, affirming that the majority of patients content.^2^**^,^**^17^ For example, Carvalho et al^8^ reported that only 26.4% of patients admitted adequately reading the IC document, while Vural et al^18^ suggested that this percentage could be even lower, potentially reaching 23%. Nevertheless, failure to read the IC document does not necessarily reflect a lack of interest in being informed. On the contrary, there is ample evidence^19^ that patients who understand their surgery, including its risks, benefits, and expected outcomes, tend to feel less stressed and report higher levels of satisfaction with their care. One study^9^ demonstrated that providing patients with detailed procedural information resulted in a 49% reduction in the number of individuals reporting high levels of anxiety. A possible explanation why patients do not read the IC form is the perception that it is just a document they need to sign, rather than a source of information about treatment.^2^ In addition, some patients believe that the primary objective of the IC process is the protection of physicians and hospitals rather than patients’ welfare.^20^ Medical staff often underestimate the information needs of their patients and merely collect signatures without explaining the essence of the IC document.^21^ We observed that presenting key information through video materials increased the patients’ awareness of the significance of the IC process and improved their understanding of the document. High-quality video materials, with animations or practical examples, effectively capture patients’ attention and encourage them to further explore the subject. In recent years, due to the widespread availability of media infrastructure and the facility of creating audiovisual contents, video-based interventions have become increasingly popular.^22^**^,^**^23^ This approach has considerable potential in patient education due to advanced visual innovations, thereby yielding substantial benefits for both patients and medical community. By integrating structured educational videos, health care providers can bridge gaps in understanding, foster greater patient confidence, and ultimately improve both patient experience and surgical outcomes.^24^**^,^**^25^ Although the primary method of transmitting information is a conversation between a health care practitioner and a patient, research has demonstrated that patients often struggle to fully comprehend information delivered through traditional methods, and frequently forget most of the verbal information received due to their educational level, age, cognitive decline, or anxiety.^26^**^,^**^27^ Video materials can trigger memory and reinforce information conveyed verbally, serving as a useful additional aid.^24^ Moreover, videos can also save surgeons time, which is currently constrained by excessive administrative responsibilities, by ensuring that patients receive standardized and comprehensive information without requiring extended individual consultations.^28^**^,^**^29^ According to patients’ opinions, video materials often provide answers to questions they intended to ask a clinician, which significantly reduces the number of topics that need to be addressed during the conversation.^24^ However, it should be emphasized that video-based interventions cannot replace a direct conversation between a medical practitioner and a patient. Literature data suggest that such negligence may also result from the existing format of IC forms.^30^**^,^**^31^ Particular concerns relate to their length and the excess of legal jargon they contain.^32^ While assessing why patients did not read their IC document, we found that the most common reason was its length, which was especially notable in the group of elderly patients. These individuals may be less inclined to read IC forms due to factors, such as decreased reading ability, reduced cognitive function, and the lack of reading glasses.^33^ Additionally, they may require more time to assimilate information, making the process more challenging, which discourages them from reading the forms.^33^ This adds to the evidence supporting a necessity of implementing changes to make the form more accessible.

Although there is ample evidence that reading the IC document lowers anxiety and improves patients’ comprehension, the results of our research do not support these findings^34^**^,^**^35^ We suspect that one of the reasons was the fact that information contained in the IC form regarding risks and possible complications was associated with increased anxiety for some individuals who read it. Moreover, just reading the IC document is not sufficient. The basis of the IC process is a surgeon-patient relationship built on mutual trust, which, in this case, seems to be crucial. We concluded that patients who did not read the IC form (majority of the CG) were better informed by health care practitioners than those who read it, which is why their axiety level was lower. Nevertheless, we believe that not providing information so as not to induce anxiety is not justified. Personal interaction remains crucial in addressing individual patient concerns and ensuring a shared decision-making process.

While evaluating various factors influencing patient engagement with IC materials, we observed significant differences in terms of surgical history, ASA status, anxiety scores, and the level of information demand on the APAIS scale in the CG. The patients without prior surgical experience were significantly more likely to read the IC form carefully and requested more details about the procedure. This could be explained by their unfamiliarity with the surgical process, leading them to seek more information to reduce uncertainty and anxiety. As indicated by previous research,^36^**^,^**^37^ the absence of prior surgical procedures is associated with an increased risk of anxiety, which may explain the observed differences in the APAIS scale results.

### Limitations

This study has certain limitations that future research could address. As it was conducted in a single medical center, its findings may not be fully generalizable to other health care settings. Larger, multicenter studies with a more diverse patient population would offer a broader perspective on the effectiveness of video-based education and external factors that may affect the readability of the document. Re-searchers should also evaluate different video-based materials with a content adjusted to facility profile, patients, and the type of IC form used.

-based materials with a content adjusted to facility profile, patients, and the type of IC form used.

## CONCLUSIONS 

The results of this study indicate the need for raising patient awareness about the importance of the IC process. Video materials are becoming an increasingly popular and effective educational tool integrating patient education with innovative visual solutions. We emphasize their remarkable potential in enhancing awareness about the significance of the IC pro-cess and strongly advocate for their integration into routine medical practice.

## JOURNAL INFORMATION

Videosurgery and Other Miniinvasive Techniques is an official journal of the Videosurgery Foundation.
